# Application of a case–control study design to investigate genotypic signatures of HIV-1 transmission

**DOI:** 10.1186/1742-4690-9-54

**Published:** 2012-06-25

**Authors:** Talia M Mota, John M Murray, Rob J Center, Damian F J Purcell, James M McCaw

**Affiliations:** 1 Department of Microbiology and Immunology, University of Melbourne, Parkville, VIC 3010, Australia; 2 Centre for Molecular, Environmental, Genetic and Analytic Epidemiology, The University of Melbourne, Parkville, VIC 3010, Australia; 3 School of Mathematics and Statistics, University of New South Wales, Sydney, NSW 2052, Australia; 4 The Kirby Institute, University of New South Wales, Sydney, NSW 2052, Australia; 5 Vaccine and Immunisation Research Group, Murdoch Childrens Research Institute, Royal Children's Hospital, Parkville, VIC 3010, Australia; 6 Melbourne School of Population Health, The University of Melbourne, Parkville, VIC 3010, Australia

**Keywords:** HIV-1, Transmission, Envelope protein, α4β7, Glycosites, Variable loops, Case–control study, Histidine, Chronic infection

## Abstract

**Background:**

The characterization of HIV-1 transmission strains may inform the design of an effective vaccine. Shorter variable loops with fewer predicted glycosites have been suggested as signatures enriched in envelope sequences derived during acute HIV-1 infection. Specifically, a transmission-linked lack of glycosites within the V1 and V2 loops of gp120 provides greater access to an α4β7 binding motif, which promotes the establishment of infection. Also, a histidine at position 12 in the leader sequence of Env has been described as a transmission signature that is selected against during chronic infection. The purpose of this study is to measure the association of the presence of an α4β7 binding motif, the number of N-linked glycosites, the length of the variable loops, and the prevalence of histidine at position 12 with HIV-1 transmission. A case–control study design was used to measure the prevalence of these variables between subtype B and C transmission sequences and frequency-matched randomly-selected sequences derived from chronically infected controls.

**Results:**

Subtype B transmission strains had shorter V3 regions than chronic strains (*p* = 0.031); subtype C transmission strains had shorter V1 loops than chronic strains (*p* = 0.047); subtype B transmission strains had more V3 loop glycosites (*p* = 0.024) than chronic strains. Further investigation showed that these statistically significant results were unlikely to be biologically meaningful. Also, there was no difference observed in the prevalence of a histidine at position 12 among transmission strains and controls of either subtype.

**Conclusions:**

Although a genetic bottleneck is observed after HIV-1 transmission, our results indicate that summary characteristics of Env hypothesised to be important in transmission are not divergent between transmission and chronic strains of either subtype. The success of a transmission strain to initiate infection may be a random event from the divergent pool of donor viral sequences. The characteristics explored through this study are important, but may not function as genotypic signatures of transmission as previously described.

## Background

Multiple research groups have invested substantial effort into defining signatures within the envelope protein (Env) of HIV-1 that are highly predictive of viral transmission [1-13]. A genetic bottleneck occurs at HIV-1 transmission, and early replicating viral populations remain homogenous [3], so that investigating the transmitted strains across many individuals may reveal a transmission signature and point to a process important in establishing infection [1,2,4-7]. These studies not only provide insight into the biological processes occurring during mucosal transmission, they may also inform the development of an effective vaccine against HIV-1. The discovery of a transmission signature could reveal motifs that may be successfully targeted during vaccination which could lead to a more protective response compared to vaccines developed from chronically replicating viral sequences [14-17].

A number of different approaches have been used to identify transmission signatures, investigating whether shorter variable loops with fewer predicted N-linked glycosylation sites (glycosites) are characteristics enriched in Env from viruses derived during acute infection. Comparing sequences from chronically infected donors and early sequences of infected recipients is a more powerful way to study transmission, although sample sizes often remain small. Using eight donor-recipient pairs, Derdeyn *et al.* showed significant differences in subtype C Env sequences, where early V1-V4 loops were shorter (*p* = 0.02) and had fewer glycosites (*p* = 0.037) [4]. Also using donor-recipient pairs, Frost *et al.* found no difference between envelope length or the number of glycosites in sequences derived from eight men who have sex with men during acute infection and their transmission pairs [18]. However, in the study Frost *et al.* state that only half of the donors had confirmed chronic infection, and that four were recently infected donor subjects [18], which could bias the results. In ten self-reported transmission pairs of subtype B, Liu *et al.* showed that gp120 sequences had shorter variable regions and reduced numbers of glycosites following transmission to the new host [19]. However, they state that their results were dependent upon how pairs were chosen for inclusion, and that no other study had yet found these reductions to be measured consistently across various acute infections. Chohan *et al.* compared 35 early and 51 late sequences of subtype A and 13 early and 82 chronic sequences of subtype B (chronic sequences were selected from the Los Alamos HIV Sequence Database (LANL) [20]). They showed early sequences had shorter loops (*p* = 0.008) and fewer glycosites (*p* = 0.017) for subtype A, but not subtype B (*p* = 0.340; *p* = 0.640, respectively) [5].

Other studies have suggested transmission-linked glycosites in the V1 and V2 loops surrounding the α4β7 binding site are significant in terms of transmission [11,15,21,22]. The V2 loop of gp120 contains an α4β7 binding site, defined as LDV/I at position 180 (according to the HXB2 reference sequence) [11,21-23]. α4β7 is a gut homing receptor colocalized with CCR5 and CD4 on CD4+ T cells, and projects out further from the cell surface than CCR5 or CD4 [11,21-24]. α4β7 +/CD4+ T cells infected with HIV-1 will migrate to the gut, where HIV-1 preferentially replicates and causes irrevocable damage to the immune system [11,21-24]. Although not necessary for HIV-1 entry into CD4+ T cells, Env lacking specific glycosites within the V1 and V2 loops of gp120 have greater access to bind α4β7, an interaction that has been shown to promote a greater susceptibility to infection [11,15,22]. This increased capacity for infection may be an important transmission signature [11,15]. However, no statistical analyses have been used to measure the association of the absence of glycans in close proximity to the α4β7 binding site and transmission.

Recently, models scanning the entire Env sequence for patterns in amino acids have been used to search for transmission signatures in subtype B Env sequences [1,2]. Gnanakaran *et al.*, through a retrospective hypothesis-raising ‘test’ analysis, found that a histidine (His) at position 12 in the Env leader sequence was associated with transmission because it was selected against in chronic infection [1,2]. Asmal *et al.* supported this phenotypically, showing that Env sequences containing a positively charged amino acid at position 12 were associated with higher levels of expression due to more efficient trafficking of the nascent polypeptide to the endoplasmic reticulum [2].

Here, we applied an epidemiological approach to investigating the transmission signature, using a case–control study design to measure associations between HIV-1 transmission strains (‘cases’) and chronic infection strains (‘controls’). Case–control studies are often used in epidemiology and measure exposure variables between cases with the outcome of interest, in this study, a transmission event, and randomly selected population controls [25,26]. The transmission strains used in this study were consensus sequences (obtained through backwards phylogeny as the most recent common ancestor of acute infection) from patients infected with one viral sequence of either subtype B or C [6,7].

Exposure variables (biological characteristics hypothesized to be associated with transmission) explored in this study were the amino acid length and number of glycosites (all PNGs are shown to be glycosylated in gp120 [27,28]) of each variable loop. Where previous studies examined the loops together [4,5], we chose to measure the hypervariable region [27] of each variable loop (see methods for loop definitions) independently to see if the biological characteristics in one loop had a significant affect where another loop did not. The numbers of glycosites in the conserved regions of Env were also explored. Other variables measured included the prevalence of the α4β7 binding site, as well as the His at position 12 in the leader sequence of Env, between transmission and chronic strains. In this study, significant associations from both a biological and statistical perspective between variables and HIV-1 transmission would be considered evidence of transmission signatures. Rather than looking for new potential transmission signatures, the aim of this study was to measure associations between previously suggested transmission signatures and their relationship with a successful transmission event.

## Results

### Amino acid lengths of the variable loops

**Figure 1:**
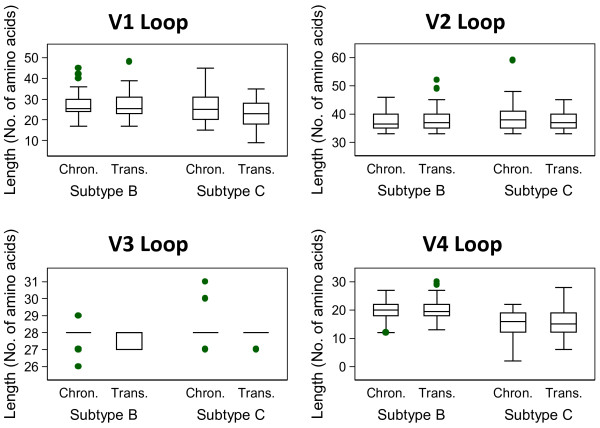
Box plots displaying amino acid length of each loop between chronic sequences and transmission strains.

When measuring the amino acid (AA) lengths, only subtype C V1 loops and subtype B V3 loops were significantly different between transmission strains and chronic controls (*p* = 0.047; *p* = 0.031, respectively; Table 1). Subtype C V1 loops were shorter in transmission strains, with a median difference of 2 AA; and the median difference between subtype B V3 lengths was zero, as demonstrated in the box plots (Figure 1). In additional analyses based on these significant findings, and because the sample size was large enough, the mean differences in amino acid length were explored. Subtype C V1 loops from transmitted strains were, on average, 3.2 AA shorter than controls; and the mean difference between V3 loops of subtype B transmission strains and controls was 0.14 AA (Additional file 1: Table S1). There were no other significant differences in AA length for the other loops or in either subtype (Figure 1).

**Table 1:** Comparison of median amino acid lengths between transmission strains and controls

		**Control median (mean) amino acid length**	**Case median (mean) amino acid length**	**Wilcoxon Rank-Sum p-value**
**V1 Loop**				
Subtype B	*n* = 78	25.5 (26.9)	25.5 (26.9)	0.87
Subtype C	*n =* 55	25 (26.8)	23 (23.5)	**0.047**
**V2 Loop**				
Subtype B	*n* = 78	36.5 (37.4)	37 (38.1)	0.292
Subtype C	*n =* 55	38 (38.8)	37 (37.6)	0.166
**V3 Loop**				
Subtype B	*n* = 78	28 (27.9)	28 (27.7)	**0.031**
Subtype C	*n =* 55	28 (28.0)	28 (27.9)	0.104
**V4 Loop**				
Subtype B	*n* = 78	20 (19.8)	19.5 (20.2)	0.829
Subtype C	*n =* 55	16 (15.5)	15 (15.5)	0.513

### Glycosites within the variable loops and conserved regions

In a logistic regression analysis exploring predictors of whether or not a strain was a transmission case or chronic control, no significant results were observed when measuring the association of the number of glycosites between cases and controls in the univariate analyses for the variable loops (Table 2) or conserved regions (Table 3). In models allowing for differences by subtype (using a statistical interaction term), odds ratios for the number of glycosites changed by over 10% in the V1 and V4 loops, but not in the V2 loop (Table 2). In the V4 loop, interaction was further confirmed by the test for homogeneity (*p* = 0.001; Table 2). This was not observed in the V1 or V2 loops. In the V3 loop, logistic regression could not be performed since all of subtype B transmission strains contained the one glycosite, as did all subtype C controls. Overall, 98.5% of transmission strains and 96.2% of controls contained this glycosite in the V3 loop (Table 4). The Wilcoxon rank sum test showed a significant difference (*p* = 0.024) between cases and controls when considering subtype B sequences with 1 versus 0 glycosites (Table 2), but the median difference of the number of glycosites in the V3 loop between transmission strains and controls was zero. There were no other significant findings in the variable loops when considering the number of glycosites, a predictor of transmission. Furthermore, there were no significant findings in the conserved regions (Table 3).

**Table 2:** Logistic Regression for predicting transmission based on number of glycosites in the variable loops

	**Univariate Odds Ratio (95% confidence interval)**	**p-value**		**Odds Ratio with subtype interaction (95% confidence interval)**	**p-value**	**Test for Homogeneity between subtype, p-value**	**Wilcoxon Rank Sum p-value**
**PNGs in V1 loop**			**PNGs in V1 loop**				
*1 to 2*			*Subtype B 1 to 2*				0.109
*3*	1.11 (0.59, 2.06)	0.751	*3*	1.42 (0.55, 3.62)	0.466		
*≥ 4*	1.62 (0.83, 3.14)	0.155	*≥ 4*	2.29 (0.83, 6.36)	0.111		
			*Subtype C 1 to 2*				0.931
			*3*	0.66 (0.18, 2.48)	0.541		
			*≥ 4*	0.54 (0.14, 2.10)	0.371	0.347	
**PNGs in V2 loop**			**PNGs in V2 loop**				
*0 to 1*			*Subtype B 0 to 1*				0.533
*2 to 3*	0.75 (0.44, 1.27)	0.282	*2 to 3*	0.84 (0.44, 1.63)	0.615		
			*Subtype C 0 to 1*				0.323
			*2 to 3*	0.67 (0.22, 2.13)	0.506	0.509	
**PNGs in V3 loop**			**PNGs in V3 loop**	*			
*0*			*Subtype B 0*				0.024
*1*	2.56 (0.49, 13.43)	0.267	*1*				
			*Subtype C 0*				0.155
			*1*			0.008	
**PNGs in V4 loop**			**PNGs in V4 loop**				
*0 to 1*			*Subtype B 0 to 1*				0.572
*2*	0.51 (0.23, 1.14)	0.102	*2*	0.36 (0.10, 1.31)	0.122		
*≥ 3*	0.54 (0.25, 1.20)	0.133	*≥ 3*	0.46 (0.13, 1.60)	0.219		
			*Subtype C 0 to 1*				0.329
			*2*	1.83 (0.35, 9.60)	0.473		
			*≥ 3*	1.19 (0.22, 6.28)	0.842	0.001	

Note: In groups where no sequences have 0 glycosites, logistic regression cannot be performed.

**Table 3:** Logistic Regression for predicting transmission based on number of conserved glycosites

	**Univariate Odd Ratio (95% confidence interval)**	**p-value**		**Odds Ratio with subtype interaction (95% confidence interval)**	**p-value**	**Test for Homogeneity between subtype, p-value**	**Wilcoxon Rank-Sum, p-value**
**PNGs in C1**			**PNGs in C1**				
*0 to 1*			*Subtype B 0 to 1*				0.538
*2*			*2*	0.78 (0.34, 1.74)	0.538		
			*Subtype C 0 to 1*				0.661
			*2*	2.63 (0.20, 34.07)	0.46	0.45	
**PNGs in C2**			**PNGs in C2**				
*0*			*Subtype B 0*				1
*1*	0.66 (0.18, 2.38)	0.522	*1*	1.0 (0.14, 7.28)	1		
			*Subtype C 0*				0.403
			*1*	0.48 (0.03, 6.74)	0.587	0.587	
**PNGs in C3**			**PNGs in C3**				
*0 to 5*			*Subtype B 0 to 5*	0.81 (0.29, 2.24)	0.68		0.669
*6*	0.76 (0.34, 1.72)	0.51	*6*	1.36 (0.50, 3.70)	0.551		
*7*	0.79 (0.36, 1.73)	0.558	*7*	0.56 (0.14, 2.26)	0.411		
*8*	0.58 (0.21, 1.62)	0.302	*8*				
			*Subtype C 0 to 5*				0.142
			*6*	0.86 (0.15, 4.85)	0.863		
			*7*	0.26 (0.05, 1.37)	0.113		
			*8*	0.90 (0.11, 7.51)	0.922	0.214	
**PNGs in C4**			**PNGs in C4**				
*0 to 3*			*Subtype B 0 to 3*				0.772
*4*	0.93 (0.51, 1.71)	0.818	*4*	1.03 (0.41, 2.58)	0.952		
*5*	079 (0.43, 1.46)	0.446	*5*	1.13 (0.48, 2.65)	0.782		
			*Subtype C 0 to 3*				0.148
			*4*	0.87 (0.25, 2.96)	0.818		
			*5*	0.23 (0.05, 1.09)	0.064	0.107	
**PNGs in C5**			**PNGs in C5**				
*0*			*Subtype B 0*				0.518
*1*	1.02 (0.25, 4.23)	0.974	*1*	0.49 (0.09, 2.78)	0.423		
*2*	0.96 (0.22, 4.06)	0.951	*2*	0.44 (0.06, 3.16)	0.413		
			*Subtype C 0*				0.951
			*1*				
			*2*			0.532	

Note: In groups where no sequences have 0 glycosites, logistic regression cannot be performed.

**Table 4:** Baseline characteristics of cases (transmission strains) and controls (chronic strains)

**Characteristics**	**Transmission Strains**	**Chronic Controls**
	**(*n* = 133)**	**(*n* = 133)**
**Subtype,*n*(%)**		
B	78 (58.7)	78 (58.7)
C	55 (41.4)	55 (41.4)
**α4β7 Binding Site,*n*(%)**		
LDV	44 (33.1)	56 (42.1)
LDI	48 (36.1)	36 (27.1)
Both	92 (69.2)	94 (70.1)
Contains the ‘D’	133 (100)	131 (98.5)
**Histidine at position 12,*n*(%)**		
	61 (45.9)	59 (44.4)
**Histidine or Arginine at position 12,*n*(%)**		
	71 (53.4)	69 (51.9)
**Amino Acid Length, V1 loop**		
Range	9 to 48	15 to 45
Mean (standard deviation)	25.5 (6.12)	26.8 (6.40)
**Amino Acid Length, V2 loop**		
Range	33 to 52	33 to 59
Mean (standard deviation)	37.9 (3.37)	38.0 (3.69)
**Amino Acid Length, V3 loop**		
Range	27 to 28	26 to 31
Mean (standard deviation)	27.8 (0.41)	27.9 (0.50)
**Amino Acid Length, V4 loop**		
Range	6 to 30	2 to 27
Mean (standard deviation)	18.2 (4.40)	18.0 (4.13)

### Exposure of the α4β7 binding site

To predict the capacity of gp120 to bind α4β7, we first determined the prevalence of this binding site in the viral sequences. Of the 133 transmission strains, 69.2% contained the LDV/I α4β7 binding site, and 100% contained the aspartic acid (D). Of the 133 controls, 70.1% contained the LDV/I, and 98.5% contained the aspartic acid at this site (Table 4). As described above, there was no difference in the number of glycosites between transmission strains and controls, and thus no difference in exposure for the binding interaction between gp120 and the α4β7 receptor on CD4+ T cells.

### Histidine at position 12

As a His at position 12 has been suggested as a transmission signature [1,2], we measured the prevalence of this motif among transmission strains and controls. 75.6% of subtype B transmission strains contained a His at position 12, as did 71.8% of the controls Additional file 1:Table S1. Using logistic regression, the odds ratio changed by over 10% from the univariate to interaction model and so subtype-specific odds ratios were reported (Table 5). There were no significant differences when measuring if a His at position 12 was a predictor of transmission. Further, the p-values from the Wilcoxon rank sum test comparing sequences with and without the His at position 12 showed no difference between transmission strains and controls for either subtype (Table 5).

**Table 5:** Logistic Regression for predicting transmission based on His at position 12.

**Univariate Odds Ratio (95% confidence interval)**	**p-value**		**Odds Ratio with subtype interaction (95% confidence interval)**	**p-value**	**Test for Homogeneity between subtype, p-value**	**Wilcoxon Rank Sum p-value**
1.06 (0.66, 0.72)	0.805	*Subtype B*	1.22 (0.60, 2.49)	0.586		0.586
		*Subtype C*	0.54 (0.08, 3.82)	0.534	0.532	0.648

## Discussion

This study found that, with one exception, there were no biologically meaningful differences in amino acid length or the number of glycosites in the variable loops or conserved regions, or the prevalence of His at position 12, between transmission strains and controls in either subtype. The exception was that the V1 loops of subtype C transmission strains were shorter than controls, based on a median difference of 2, and a mean difference of 3.2 AA. Our results, derived from a case–control study design with the largest currently available data set (133 “founder” sequences and matched controls) indicate that if there is indeed a “transmission signature” for HIV-1 then it is not manifest in the previously hypothesized summary measures that we have investigated.

As in all case–control studies, we cannot exclude potential confounding effects due to unrecorded factors such as route of transmission, coinfection with other STIs, or phylogenetic relationships between samples [25,26]. Another limitation is that during the random control selection, it is possible some transmission strains may have been selected. Such selections, expected to be exceedingly unlikely due to the diversity of circulating viral sequences within an individual, would bias our results towards the null hypotheses of no difference between transmission and chronic strains. Another potential limitation of this study is that the sequences used for cases and controls have not been validated for functionality. This is an issue that is inherent in studies examining large numbers of sequences at a population level. Other information that would greatly benefit investigations of this type include the sequence variation of viruses in the transmitting fluids of the infected donor, and how variation from this quasispecies decreases in the new host at the mucosa and through early selection pressure to persist beyond this genetic bottleneck. Unfortunately this information is currently unobtainable.

Patients that displayed multivariant transmission were excluded from this study because it is currently unknown what factors lead to multiple viruses establishing infection. Rieder *et al.* investigated envelope sequences from 145 patients during acute infection. They found that concomitant STI, gender, and sexual practice were not associated with transmission of heterogeneous virus populations, suggesting that transmission of multiple HIV-1 variants is not dependent on mucosal factors, but a more complex combination of factors that have yet to be determined [29].

Current research shows that having a His at position 12 in the Env leader sequence is enriched in transmission strains [1,2]. However, our study showed there was no difference in the prevalence of His between transmission strains and controls of subtype B or C. Asmal *et al.* also indicated that His is the most common AA at position 12 in subtype B and most other subtypes [2]. However, our study revealed that the overall prevalence of His at position 12 in subtype C is small (4.5%; Additional file 2: Table S2), whilst glutamine, an AA without charge, is most common at position 12 among transmission strains and controls of subtype C (85.5% and 76.4%, respectively).

Gnanakaran *et al.* showed that consensus sequences of Subtype B transmission strains contained a His at position 12 more often than consensus sequences of chronic strains (74% versus 57%) [1]. Of note, in this study, there was no statistical difference between transmission strains and controls of subtype B (72% versus 76%; Additional file 2: Table S2). The differences between the prevalence of His at position 12 in these chronic populations could be due to control selection where we selected controls from the Los Alamos database (*n* = 78) and Gnanakaran *et al.* used intra-patient consensus sequences that had all been sequenced using single genome amplification (*n* = 43) [1]. However, in their study, Gnanakaran *et al.* also used another set of controls from chronically infected patients selected from the Los Alamos database to increase their statistical power [1]. After investigation of this control population, we found that 86% of these chronic sequences contained a His at position 12, supporting our findings.

The various methods used in this study allow different statistical parameters to be explored. This is advantageous when searching for biological meaning behind statistical significance. The Wilcoxon rank sum test concludes a significant difference in subtype B V3 AA length by determining the probability that differences in median length occurred by chance between transmission strains and controls from chronic infection. However, further investigation reveals that the median difference is zero. This is also reflected in the *t*-test (where we probe mean, rather than median) where we detected that on average, the V3 loops of transmission strains are just 0.14 AA shorter. This value does not represent a full AA, and therefore the means of the groups are biologically similar, if not indistinguishable. This deeper investigation enables us to interpret biological meaning, rather than just presenting significant p-values from the Wilcoxon rank sum test. In subtype C, the V1 loops of transmission strains are on average, 3.2 AA acids shorter than chronic controls. While this is a more relevant number consisting of greater than one residue, phenotypic studies must consider if three residues contributes to a functional difference in envelope behavior that would be important during transmission. There were no biologically relevant differences in the number of glycosites between transmission strains and chronic controls for either subtype.

## Conclusions

When gp120 has access to bind α4β7, there exists a capacity for a population of CD4+ T cells to be highly susceptible to infection with HIV-1 [11,15,21-23]. However, no differences in the number of glycosites in the V1 and V2 loops were observed between transmission strains and controls of either subtype. These results suggest that although sequences lacking these glycosites can bind α4β7, it may not be an event strongly associated with transmission. Likewise, the His at position 12 in the leader sequence does impact envelope expression and thus virion infectivity [1,2]. However, the results from this study suggest the prevalence of His is not different between transmission strains and controls.

Although important, the characteristics explored through this study may not function as genotypic signatures characteristic of HIV-1 during transmission, and may not differ throughout pathogenesis. Although a genetic bottleneck is observed after transmission, the selection of the transmitting strain from the divergent pool of donor viral sequences may be a random event. The “random transmission” of HIV-1 has been hypothesized by Hedskog *et al.*, who provide a compelling argument that in the context of CCR5, CXCR4, and R5X4 coreceptor use at transmission, the viral characteristics that dominate the viral population of the donor will be transmitted [30].

Because of discordant findings across research studies, further genotypic, phenotypic, and structural studies with larger sample sizes are warranted and may require the exploration of the subtypes independently for vaccine design.

## Methods

To investigate genotypic signatures of HIV-1 transmission, a case–control study design was used to measure the association of AA lengths and the number of glycosites in each of the variable loops of gp120, as well as the conserved regions. The prevalence of the α4β7 binding site (LDV/I) was also investigated due to the transmission-linked associations of glycosites surrounding this site in the V2 loop of gp120 [11,21-23]. In addition, the prevalence of a His at position 12 was determined, and the association of this residue as a transmission signature was measured. The total sample size of 266 sequences was powered at 80% to reject the null hypothesis (of no difference) at 95% precision.

133 transmission strain cases (78 subtype B, 55 subtype C) were acquired from Keele *et al.* and Abrahams *et al.*[6,7]*.* These envelope sequences, derived during acute infection, were obtained from individuals infected with only one transmitting virus, as determined using a mathematical model of backwards phylogeny [6,7]. Keele *et al.* and Abrahams *et al.* were able to define the most recent common ancestor of infection (the consensus founder sequence) for each case and it is these sequences that are used as the transmission strains in this study. To date, only Keele *et al.* and Abrahams *et al.* have defined transmission strains in this way. Therefore, we have used the entire founder “case population” currently available for this type of study.

**Figure 2:**
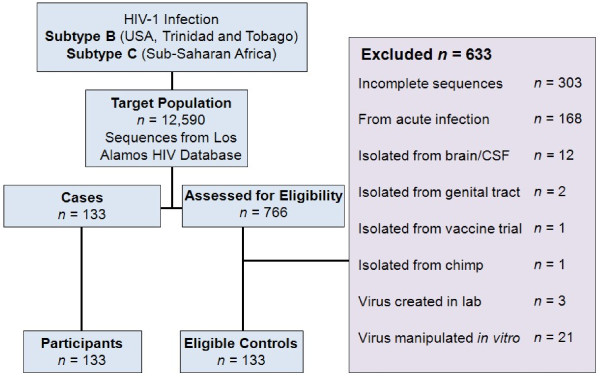
Case and Control Selection.

133 control sequences were derived from the plasma of individuals with chronic infection, acquired from LANL [20]. A random numbers table was generated using the Stat Trek Random Number Generator http://stattrek.com to guarantee the random selection of controls. Each selected control sequence was investigated and submitted to rigorous exclusion criteria, contributing to the strong internal validity of this study (Figure 2). For example, if a randomly selected sequence happened to come from a patient whom we had already derived a control sequence from, that newly selected sequence was discarded and another random selection from the LANL database made. This process ensured the statistical independence of our control samples and that all sequences from a particular patient in the LANL database had the same chance of being selected as a control sequence. To adjust for confounding variables, controls were frequency-matched on HIV-1 subtype and geographical location. However, only the subtype designation was considered in the analyses due to the collinearity between location and subtype. 78 subtype B chronic infection controls were selected from the USA/Trinidad and Tobago and 55 subtype C sequences were selected from South Africa/Malawi. Demographic details including behaviour status were not available for most control sequences and so were not controlled for in the analyses.

Case and control DNA sequences, as well as the HXB2 reference envelope sequence [27], were converted to AA sequences and then aligned using a progressive multiple alignment method (multialign, MATLAB 2010b, The MathWorks Inc., Natick MA, USA). AA positions in the aligned sequences were numbered relative to HXB2 according to the convention of Korber *et al.*[31]*.* N-glycosylation sites were then determined on the original sequence for each variable loop identified through the alignment. LDV/I residues and position 12 Histidine residues were counted in each of the 266 sequences. AA lengths and glycosites were counted separately for each of the variable loops (V1-V4) and conserved regions. In this study, the hypervariable regions of the gp120 variable loops were used and defined as Env137-151 for the V1 loop, Env161-195 for the V2 loop, Env300-328 for the V3 loop, and Env393-414 for the V4 loop according to the HXB2 sequence [27].

The Wilcoxon rank sum test was used to determine differences in the AA lengths among the variable loops between cases and controls, analysed separately for subtype B and subtype C. The null hypothesis of the rank sum test was that when values are ranked in ascending order, there is no difference in median values between both groups. Box plots were created to present the observations. Where results displayed p-values less than or equal to 0.05, t-tests were also performed to explore the mean difference.

**Figure 3:**
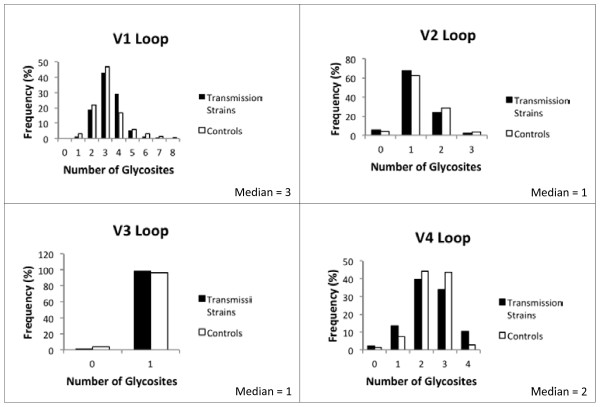
** Histograms of baseline frequencies of the number of glycosites in each variable loop (for subtype-specific characteristics, see Additional file **2**: Table S2).**

Logistic regression was used to assess the association of the number of glycosites on transmission. The number of glycosites was considered a categorical variable, based on the distribution and median values of the number of glycosites in each variable loop and each conserved region among the sequences (Figure 3; characteristics for conserved regions not shown). For example, the V1 and V4 loops have the largest number of glycosites present (1 to 8 and 0 to 4, respectively); the median value is an independent category, with categories above and below the median value. The range in the V2 loop (0 to 3) is smaller. The median value is included in the lower category and values above the median are in a higher category. Both cases and controls have either 0 or 1 glycosite in the V3 loop.

Because subtypes are genetically distinct, subtype specific odds ratios were measured in a model that tested for interaction between the subtypes. When compared to the univariate models, a change in odds ratio greater than 10% for either subtype was used as a condition to include the interaction and report both univariate and subtype-specific odds ratios (Table 2). The null hypotheses were that the number of glycosites is not associated with HIV-1 transmission in any loop or conserved region, and that subtype does not modify this effect (interaction).

Logistic regression was used to assess the relationship between the His at position 12 and transmission. The null hypothesis with logistic regression was that a sequence containing a His at position 12 is not associated with HIV-1 transmission, and that subtype does not affect this association.

For both logistic models, Wilcoxon rank sum tests were used to test for differences in the number of glycosites and the presence of His at position 12 for comparison with previously published work. All analyses were performed using Stata version 10.1 (Stata Corp, TX, USA).

## Abbreviations

PNGs, predicted N-linked glycosylation sites; glycosites; LANL, Los Alamos National Laboratory; HIV, Sequence Database; STI, sexually transmitted infection.

## Competing interests

The authors declare that they have no competing interests.

## Author’s contributions

TM designed the study, collected the data, performed the statistical analyses, interpreted the data and prepared the manuscript. JMM aligned the sequences and participated in data collection and provided helpful discussion regarding data analysis. RJC provided meaningful discussion and helped to interpret the data. DFJP conceived of the virological aspects of the study and provided helpful discussion. JMcC oversaw and contributed to the design of the study and helped to draft the manuscript. All authors read and approved the final manuscript. Both corresponding authors contributed equally to this paper.

## **Accession Numbers**

Control sequences: all from **LANL** [AY352275, AY357342, EF593188, DQ853447, DQ410638, DQ410615, DQ410601, DQ410579, DQ410566, DQ410557, DQ410531, DQ410522, DQ410509, DQ410474, DQ410422, DQ410400, DQ410361, DQ410321, DQ410275, DQ410237, DQ410224, DQ410214, DQ410170, DQ410119, GU728030, DQ410096, DQ410083, DQ410058, AY835781, AY835775, AY835771, AY835763, AY835759, AY842843, AY842821, AY842807, U69584, AF025763, AF025760, AF025750, U26546, FJ469771, FJ469768, FJ469755, FJ469749, FJ469746, FJ469735, FJ469732, FJ469712, FJ469700, FJ653616, FJ653586, FJ653567, FJ653545, FJ653486, FJ653473, FJ653427, FJ653397, FJ653378, FJ653309, FJ653240, FJ653208, FJ653135, FJ653113, FJ798580, FJ798423, FJ798397, GU938295, GU727972, GU728371, EU604604, GU728337, EU604554, GU728368, GU728375, GU728244, GU728195, GU728369, AB485644, AF110967, AF110977, AF290027, AF391244, AF391246, AF443087, AF443097, AF443099, AY463234, AY529660, AY772691, AY878056, AY901970, AY901972, DQ093591, DQ164122, DQ275658, DQ275660, DQ351224, DQ351233, DQ369982, DQ369984, DQ382365, DQ382379, DQ396367, DQ396377, DQ396399, DQ396399, DQ978981, FJ846650, GU080179, GU216703, GU216785, GU216805, GU216824, GU216838, GU329068, GU329085, GU329098, GU329118, GU329147, GU329160, GU329169, GU329191, GU329208, GU329221, GU329241, GU329261, GU329273, GU329294, GU329314, GU329377, GU329389, GU329409]

Transmission strain consensus sequences: Obtained through personal correspondence with Brandon F. Keele and Melissa-Rose Abrahams.

## Supplementary Material

Additional file 1 **Table S1.** T-tests performed on groups resulting in significant findings from Table 1. Click here for file

Additional file 2 **Table S2.** Subtype-specific baseline characteristics. Click here for file
