# Supplementary material for: Application of a case–control study design to investigate genotypic signatures of HIV-1 transmission
Source: Retrovirology. 2012 Jun 25;9:54. doi: 10.1186/1742-4690-9-54 (PMC3419081; doi:10.1186/1742-4690-9-54)
Supplement: Additional file 1 — Table S1. T-tests performed on groups resulting in significant findings from Table 1. [file 1742-4690-9-54-S1.doc]

**Table S1.** T-tests performed on groups resulting in significant findings from Table 1.

|  | **Median** | **Mean** | **Mean Difference (95% confidence interval)** | **t-test p-value** |
| --- | --- | --- | --- | --- |
| **Subtype C V1 Loop**  *Transmission Strains* | 23 | 23.5 |  |  |
| *Chronic Controls* | 25 | 26.8 | 3.2 (0.57, 5.86) | 0.018 |
| **Subtype B V3 Loop** |  |  |  |  |
| *Transmission Strains* | 28 | 27.7 |  |  |
| *Chronic Controls* | 28 | 27.9 | 0.14 (0.01, 0.28) | 0.048 |
