# Supplementary material for: Application of a case–control study design to investigate genotypic signatures of HIV-1 transmission
Source: Retrovirology. 2012 Jun 25;9:54. doi: 10.1186/1742-4690-9-54 (PMC3419081; doi:10.1186/1742-4690-9-54)
Supplement: Additional file 2 — Table S2. Subtype-specific baseline characteristics. [file 1742-4690-9-54-S2.doc]

**Table S2.** Subtype-specific baseline characteristics

| **Characteristics** | **Transmission Strains** | **Chronic Controls** |
| --- | --- | --- |
|  | (*n* = 133) | (*n* = 133) |
| **Subtype, *n*(%)** |  |  |
| B | 78 (58.7) | 78 (58.7) |
| C | 55 (41.4) | 55 (41.4) |
| **α4β7 Binding Site, *n*(%)** |  |  |
| *Subtype B*  LDV | 39 (50.0) | 47 (60.3) |
| LDI | 23 (29.5) | 16 (20.5) |
| Both | 62 (79.5) | 63 (80.8) |
| Contains the ‘D’ |  |  |
| *Subtype C*  LDV | 5 (9.1) | 9 (16.4) |
| LDI | 25 (45.5) | 20 (36.4) |
| Both | 30 (54.5) | 29 (52.7) |
| Contains the ‘D’ |  |  |
| **Histidine at position 12, *n*(%)** |  |  |
| *Subtype B* | 59 (75.6) | 56 (71.8) |
| *Subtype C* | 2 (3.6) | 3 (5.5) |
| **Histidine or Arginine at position 12, *n*(%)** | |  |
| *Subtype B* | 65 (83.3) | 62 (79.5) |
| *Subtype C* | 6 (10.9) | 7 (12.7) |
| **Amino Acid Length, V1 loop** |  |  |
| *Subtype B* |  |  |
| Range | 17 to 48 |  |
| Mean (standard deviation) | 26.9 (5.6) | 26.9 (5.4) |
| *Subtype C* |  |  |
| Range | 9 to 45 |  |
| Mean (standard deviation) | 23.5 (6.3) | 26.8 (7.7) |
| **Amino Acid Length, V2 loop** |  |  |
| *Subtype B* |  |  |
| Range | 33 to 52 |  |
| Mean (standard deviation) | 38.1 (3.6) | 37.4 (3.0) |
| *Subtype C* |  |  |
| Range | 33 to 59 |  |
| Mean (standard deviation) | 37.6 (2.9) | 38.8 (4.4) |
| **Amino Acid Length, V3 loop** |  |  |
| *Subtype B* |  |  |
| Range | 26 to 29 |  |
| Mean (standard deviation) | 27.7 (0.5) | 27.9 (0.4) |
| *Subtype C* |  |  |
| Range | 27 to 31 |  |
| Mean (standard deviation) | 27.9 (0.4) | 28.0 (0.6) |
| **Amino Acid Length, V4 loop** |  |  |
| *Subtype B* |  |  |
| Range | 12 to 30 |  |
| Mean (standard deviation) | 20.2 (3.2) | 19.8 (2.8) |
| *Subtype C* |  |  |
| Range | 2 to 28 |  |
| Mean (standard deviation) | 15.5 (4.4) | 15.5 (4.4) |
| **V1 loop: total # of glycosites, *n*(%)** |  |  |
| *Subtype B* |  |  |
| Median # of Glycosites = 3 |  |  |
| 0 glycosites | 0 | 0 |
| 1 glycosite | 0 | 1 (1.3) |
| 2 glycosites | 9 (11.5) | 13 (16.7) |
| 3 glycosites | 41 (52.6) | 45 (57.7) |
| 4 glycosites | 22 (28.2) | 13 (16.7) |
| 5 glycosites | 3 (3.8) | 3 (3.8) |
| 6 glycosites | 2 (2.6) | 0 |
| 7 glycosites | 1 (1.3) | 2 (2.6) |
| 8 glycosites | 0 | 1 (1.3) |
| **V1 loop: total # of glycosites, *n*(%)** |  |  |
| *Subtype C* |  |  |
| Median # of Glycosites = 3 |  |  |
| 0 glycosites | 0 | 0 |
| 1 glycosite | 2 (3.6) | 3 (5.5) |
| 2 glycosites | 16 (29.0) | 16 (29.0) |
| 3 glycosites | 16 (29.0) | 18 (32.7) |
| 4 glycosites | 17 (30.1) | 9 (16.4) |
| 5 glycosites | 4 (7.3) | 5 (9.0) |
| 6 glycosites | 0 | 4 (7.3) |
| 7 glycosites | 0 | 0 |
| 8 glycosites | 0 | 0 |
| **V2 loop: total # of glycosites, *n*(%)** |  |  |
| *Subtype B* |  |  |
| Median # of glycosites = 1 |  |  |
| 0 glycosites | 2 (2.6) | 0 |
| 1 glycosite | 50 (64.1) | 49 (62.8) |
| 2 glycosites | 23 (29.5) | 27 (34.6) |
| 3 glycosites | 3 (3.8) | 2 (2.6) |
| *Subtype C* |  |  |
| Median # of glycosites = 1 |  |  |
| 0 glycosites | 6 (7.7) | 6 (7.7) |
| 1 glycosite | 40 (51.3) | 35 (44.9) |
| 2 glycosites | 9 (11.5) | 11 (14.1) |
| 3 glycosites | 0 | 3 (3.8) |
| **V3 loop: total # of glycosites, *n*(%)** |  |  |
| *Subtype B* |  |  |
| Median # of glycosites = 1 |  |  |
| 0 glycosites | 0 | 5 (6.4) |
| 1 glycosite | 78 (100) | 73 (93.6) |
| **V3 loop: total # of glycosites, *n*(%)** |  |  |
| *Subtype C* |  |  |
| Median # of glycosites = 1 |  |  |
| 0 glycosites | 2 (3.6) | 0 |
| 1 glycosite | 53 (96.4) | 55 (100%) |
| **V4 loop: total # of glycosites, *n*(%)** |  |  |
| *Subtype B* |  |  |
| Median # of glycosites = 3 |  |  |
| 0 glycosites | 1 (1.3) | 0 |
| 1 glycosite | 8 (10.3) | 4 (5.1) |
| 2 glycosites | 27 (34.6) | 33 (42.3) |
| 3 glycosites | 31 (39.7) | 40 (51.3) |
| 4 glycosites | 11 (14.1) | 1 (1.3) |
| **V4 loop: total # of glycosites, *n*(%)** |  |  |
| *Subtype C* |  |  |
| Median # of glycosites = 2 |  |  |
| 0 glycosites | 2 (3.6) | 2 (3.6) |
| 1 glycosite | 10 (18.2) | 6 (10.9) |
| 2 glycosites | 26 (47.3) | 26 (47.3) |
| 3 glycosites | 14 (25.5) | 18 (32.7) |
| 4 glycosites | 3 (5.5) | 3 (5.5) |
| **C1: total # of glycosites, *n*(%)** |  |  |
| *Subtype B* |  |  |
| Median # of Glycosites = 1 |  |  |
| 0 glycosites | 0 | 0 |
| 1 glycosite | 65 (83.3) | 62 (79.5) |
| 2 glycosites | 13 (16.7) | 16 (20.5) |
| **C1: total # of glycosites, *n*(%)** |  |  |
| *Subtype C* |  |  |
| Median # of Glycosites = 1 |  |  |
| 0 glycosites | 2 (3.6) | 0 |
| 1 glycosite | 51 (9.3) | 54 (98.2) |
| 2 glycosites | 2 (3.6) | 1 (1.8) |
| **C2: total # of glycosites, *n*(%)** |  |  |
| *Subtype B* |  |  |
| Median # of glycosites = 1 |  |  |
| 0 glycosites | 2 (2.6) | 2 (2.6) |
| 1 glycosite | 76 (97.4) | 76 (97.4) |
| **C2: total # of glycosites, *n*(%)** |  |  |
| *Subtype C* |  |  |
| Median # of glycosites = 1 |  |  |
| 0 glycosites | 4 (7.2) | 2 (3.6) |
| 1 glycosite | 51 (92.7) | 53 (96.4) |
| **C3: total # of glycosites, *n*(%)** |  |  |
| *Subtype B* |  |  |
| Median # of glycosites = 7 |  |  |
| 0 glycosites | 0 | 0 |
| 1 glycosite | 0 | 0 |
| 2 glycosites | 0 | 0 |
| 3 glycosites | 0 | 2 (2.6) |
| 4 glycosites | 10 (12.8) | 8 (10.3) |
| 5 glycosites | 25 (32.1) | 31 (39.7) |
| 6 glycosites | 38 (48.7) | 28 (35.9) |
| 7 glycosites | 5 (6.4) | 9 (11.5) |
| 8 glycosites | 0 | 0 |
| **C3: total # of glycosites, *n*(%)** |  |  |
| *Subtype C* |  |  |
| Median # of glycosites = 7 |  |  |
| 0 glycosites | 0 | 1 (1.8) |
| 1 glycosite | 0 | 0 |
| 2 glycosites | 0 | 0 |
| 3 glycosites | 0 | 0 |
| 4 glycosites | 0 | 0 |
| 5 glycosites | 8 (14.5) | 3 (5.5) |
| 6 glycosites | 18 (32.7) | 13 (23.6) |
| 7 glycosites | 22 (40.0) | 31 (56.4) |
| 8 glycosites | 7 (12.7) | 7 (12.7) |
| **C4: total # of glycosites, *n*(%)** |  |  |
| *Subtype B* |  |  |
| Median # of glycosites = 4.5 |  |  |
| 0 glycosites | 0 | 0 |
| 1 glycosite | 0 | 0 |
| 2 glycosites | 2 (2.6) | 1 (1.3) |
| 3 glycosites | 12 (15.4) | 14 (17.9) |
| 4 glycosites | 24 (30.8) | 25 (32.1) |
| 5 glycosites | 40 (51.3) | 38 (48.7) |
| **C4: total # of glycosites, *n*(%)** |  |  |
| *Subtype C* |  |  |
| Median # of glycosites = 4 |  |  |
| 0 glycosites | 0 | 1 (1.8) |
| 1 glycosite | 0 | 1 (1.8) |
| 2 glycosites | 4 (7.3) | 3 (5.5) |
| 3 glycosites | 20 (36.4) | 14 (25.5) |
| 4 glycosites | 27 (49.1) | 24 (43.6) |
| 5 glycosites | 4 (7.3) | 12 (21.8) |
| **C5: total # of glycosites, *n*(%)** |  |  |
| *Subtype B* |  |  |
| Median # of glycosites = 1 |  |  |
| 0 glycosites | 4 (5.1) | 2 (2.6) |
| 1 glycosite | 67 (85.9) | 68 (87.2) |
| 2 glycosites | 7 (9.0) | 8 (10.3) |
| **C5: total # of glycosites, *n*(%)** |  |  |
| *Subtype C* |  |  |
| Median # of glycosites = 2 |  |  |
| 0 glycosites | 0 | 2 (3.6) |
| 1 glycosite | 19 (34.5) | 16 (29.1) |
| 2 glycosites | 36 (65.5) | 37 (67.3) |
